# Is COVID-19 more severe in older men?

**DOI:** 10.1136/postgradmedj-2020-137867

**Published:** 2020-05-13

**Authors:** Xiaopeng Liang

**Affiliations:** 1 Medicine, University of Hong Kong, Hong Kong, Hong Kong; 2 National Center for Cardiovascular Diseases, Fuwai Hospital, Chinese Academy of Medical Sciences & Peking Union Medical College, Xicheng District, Beijing, China

In the past 3 months, the pandemic caused by COVID-19 has been a considerable threat facing the entire world, causing substantial morbidity and mortality. By 20 April 2020, a total of 2 285 210 cases of positive laboratory-confirmed COVID-19 cases had been reported worldwide, and 155 124 deaths had been documented in over 200 territories. The worldwide mortality rate is around 4.5%,^1^ although the actual mortality rate is probably lower due to undiagnosed cases. Previous studies on the severe acute respiratory syndrome coronavirus (SARS-CoV) and the Middle East respiratory syndrome coronavirus (MERS-CoV) showed that older men were at higher risk of coronavirus infection. Older men also seem to be more susceptible to COVID-19. Furthermore, patients older than 65 years with comorbidities have poorer outcomes. In a study of 1099 COVID-19 patients, the median age of patients with severe disease (173/1099) was 7 years higher than that of those with non-severe disease (926/1099). The mortality ratio of males to females was 3.25.^2^ Studies in female mice suggested that oestrogen signalling can directly inhibit the replication of SARS-CoV, thereby protecting the mice from infection.^3^ Oestrogen level varies with age, rising in prepubertal patients and declining with age. Thus, age-associated decrease in oestradiol concentration might be a possible explanation for the susceptibility and severe progression of COVID-19 in older patients.

The immunopathology of COVID-19 is still unclear. In SARS, high levels of pro-inflammatory cytokines including Monocyte chemoattractant protein-1, interleukin (IL)-2, IL-5, IL-10, granulocyte-macrophage colony-stimulating factor, interferon γ-induced protein 10 kDa and macrophage inflammatory protein-1 alpha were found, resulting in acute lung injury and multi-organ dysfunction.^4^ While a robust immune system is needed to protect against viral infection, an excessive response may be detrimental. By analogy with SARS and MERS, lymphopenia and cytokine storm might play a role in the deterioration of COVID-19 patients. There is a sex difference in immune response that is well-known but poorly understood. Females react more aggressively to antigens, including self-antigens. It is tempting to speculate that they could mount a better immune response to the coronavirus. Conversely, older patients and men are not protected by oestrogens.

Like SARS-CoV, SARS-CoV2 replicates mainly in the respiratory tract and alveolar epithelium. SARS-CoV2, the virus responsible for COVID-19, is known to gain entry into cells via the angiotensin-converting enzyme 2 (ACE2) receptor. This may also be the mechanism behind cardiac and renal dysfunction in COVID-19. Oestrogen suppresses the cardiac renin-angiotensin system and reduces lung and myocardial ACE2 expression.^5^ Lower oestrogen levels increase ACE2 expression, facilitating the development of cardiovascular diseases and hypertension. Whether increase in ACE2 and its receptor increases susceptibility to COVID-19 is uncertain. In a study of 1099 COVID-19 patients, 23.7% of the severe cases suffered from hypertension and 16.2% suffered from diabetes, whereas in the non-severe group, only 13.4% had hypertension and 5.7% had diabetes.^2^

The increased susceptibility of older people and men may also be explained by the different smoking rates. In pulmonary inflammation induced by smoking, there are continuous accumulation of leucocytes and secretion of pro-inflammatory cytokines such as tumor necrosis factor alpha, IL-6, IL-8 and granulocyte-macrophage colony-stimulating factor, resulting in damage to lung mucosa and bronchi.^4^ Chronic smokers are prone to pulmonary infections. Moreover, nicotine increases the expression of ACE2.

As the number of COVID-19 patients increases, our understanding of its pathology and epidemiology will improve correspondingly. Identifying the risk factors for COVID-19 infection and complications is important, as it will help us to hospitalise and treat high-risk patients promptly.

## References

[1] World Health Organization Coronavirus disease (COVID-19) outbreak. Available: https://www.who.int/emergencies/diseases/novel-coronavirus-2019

[2] Guan W-jie, Ni Z-yi, Hu Y, et al Clinical characteristics of coronavirus disease 2019 in China. N Engl J Med Overseas Ed 2020 10.1056/NEJMoa2002032 PMC7092819 32109013

[3] Robinson DP, Hall OJ, Nilles TL, et al 17β-estradiol protects females against influenza by recruiting neutrophils and increasing virus-specific CD8 T cell responses in the lungs. J Virol 2014;88:4711–20. 10.1128/JVI.02081-13 24522912 PMC3993800

[4] Nicholls JM, Poon LLM, Lee KC, et al Lung pathology of fatal severe acute respiratory syndrome. Lancet 2003;361:1773–8. 10.1016/S0140-6736(03)13413-7 12781536 PMC7112492

[5] Wang H, Jessup JA, Zhao Z, et al Characterization of the cardiac renin angiotensin system in oophorectomized and estrogen-replete mRen2.Lewis rats. PLoS One 2013;8:e76992.. 10.1371/journal.pone.0076992 24204720 PMC3808369

